# The effect of trait mindfulness on social media rumination: Upward social comparison as a moderated mediator

**DOI:** 10.3389/fpsyg.2022.931572

**Published:** 2022-10-04

**Authors:** Chenyu Gu, Shiyu Liu, Subai Chen

**Affiliations:** School of Journalism and Communication, Xiamen University, Xiamen, China

**Keywords:** mindfulness, social media rumination, upward social comparison, self-esteem, social media

## Abstract

Social media rumination means that social media users are inclined to worry about their posts, relevant situational elements, and ramifications of the posts on a regular basis, and it is one of the main reasons why people's use of social media is linked to unfavorable mental health and interpersonal results. Rumination is antagonistic to mindfulness, which entails paying attention on purpose and without judgment, and mindfulness appears to be useful in reducing rumination. However, in the context of social media, the nature of the relationship between rumination and mindfulness has gained less attention. The current research study indicates that trait mindfulness, upward social comparison (USC), and self-esteem are implicated in social media rumination (SMR). However, no research study has synthesized the findings into one model. Therefore, the current research study aims to explore the relationship between trait mindfulness and SMR, the mediating effect of USC, and the moderating effect of self-esteem. The model was tested using AMOS and the PROCESS macro in SPSS with bootstrapping. According to the findings, SC appears to have a moderated mediator effect on the relationship between trait mindfulness and SMR. Specifically, individuals with high mindfulness trait do not necessarily have less USC. Self-esteem moderated the above mediation. The beneficial effect of trait mindfulness on social media rumination is explained in depth in this study.

## Introduction

Mobile applications and websites known as “social media” used by individuals to create and share ideas or feelings influence the lifestyle and behavior of each individual to some degree. Recent studies have linked the use of social media with increase in depressive symptoms and suicide rates, calling for a better perception of the elemental process of the effect of social media (Twenge et al., 2019). As rumination is a significant factor in maintaining and even exacerbating psychological distress (Nolen-Hoeksema and Morrow, 1993), social media rumination, which is the tendency to obsessively worry and brood over social media posts, may also be an important indicator of psychological health, but most previous research studies on this topic have relied on broad rumination measures (Parris et al., 2020). The specificity of social media rumination may help us better explain the negative effect related to social media and provide feasible suggestions to deal with them. Protective factors reducing the risk of rumination in social media context are scarcely studied.

Research on mindfulness is a relatively new topic in psychology, also leading to increase in analyzing mindfulness training in the West from the 1970's (Keng et al., 2011). Mindfulness is also considered a positive personality trait. Dispositional mindfulness, as a personality trait, has been proved to be related to generation of human emotions (Kong, 2015). Growing evidence suggests that rumination can be significantly restrained by dispositional mindfulness, which refers to the awareness of and sustained attentiveness to one's present moment without any judgment (Raes and Williams, 2010; Heeren and Philippot, 2011). It should be emphasized that this study focuses on mindfulness as a personality trait rather than mindfulness training. Consequently, we infer that there would be a similar relationship between trait mindfulness and social media rumination. However, few studies have empirically investigated the mechanism of the influence of mindfulness on rumination in social media context. In addition, with the aim of better understanding the relationship between mindfulness and social media rumination, this study introduces the upward social comparison, which has been found to be associated with negative psycho-emotional wellbeing (Jang et al., 2016), rumination (Yang et al., 2021), and mindfulness (Langer et al., 2010), as the mediation variable. As the effect of mindfulness on social comparison is moderated by other variables (Langer et al., 2010), such as self-esteem, an attitude that each individual has toward himself (Coopersmith, 1967) and is regarded as an important moderation variable (Apaolaza et al., 2019). Previous research has shown that mindfulness is more effective in reducing harmful reactions to events in individuals with different levels of self-esteem, with mindfulness tending to be more effective in individuals with low self-esteem (Ford, 2017). Therefore, we incorporated self-esteem into our conceptual model.

Previous research studies have suggested that mindfulness is related to social media rumination, whereas if and how upward social comparison and self-esteem play a role demands further research and discussion. The present study would make contribution in theory in two ways. First, as some outcome factors are rarely explored in social media context, this study focuses on them, among which and in particular, social media rumination is selected as the main outcome variable. As the use of social media could easily lead to a mental problem for individuals, this research is aimed at exploring whether trait mindfulness could be an approach to reduce this negative effect. Second, this study contributes to the extant research by investigating how trait mindfulness influences upward comparison and rumination in the social media context. The findings of present research study can help us have a better understanding of how trait mindfulness can help individuals to avoid or reduce social media rumination through the process of upward social comparison's mediating effect and moderation of self-esteem, consequently leading to a better online experience.

## Theoretical framework

### Mindfulness

The concept of mindfulness arises from the meditation training in Buddhism and refers to the intensity of attention (Jacobs and Blustein, 2008). It can be viewed as a stable psychological state and requires people to consciously focus on a fixed object and be attentive to the present. Jon Kabat Zinn applied it to clinical therapy and psychotherapy. Due to its remarkable treatment effect and universal applicability, the application of mindfulness intervention has developed rapidly and attracted more and more attention. In addition to “awareness,” “attention,” and “remembering,” researchers add “nonjudgment” and “acceptance” to the concept of mindfulness (Kabat-Zinn, 2003). In short, it could be referred to as awareness of the situation combined with the attitude of no judging of any thoughts or feelings (Keng et al., 2011). In research, mindfulness is generally divided into state mindfulness and trait mindfulness (Randal et al., 2015). Trait mindfulness refers to one's ability to focus on what is happening at the present moment, and it can be seen as a stable state of mind that requires one to consciously focus on a fixed object and pay attention to the present moment. In brief, it can be referred to as awareness of the situation and a non-judgmental attitude toward any thought or feeling (Keng et al., 2011). To explore sustained rather than transient behavioral effects, the present study focused on trait mindfulness.

Over the past few years, the benefits of mindfulness have received more attention in research. It has been found that mindfulness can bring benefits to individuals such as: maintaining mental health (Brown and Ryan, 2003), increasing subjective well-being (Liu et al., 2020a), enhancing intensity of attention and mental lucidity (Hanley and Garland, 2017), increasing productivity (Liu et al., 2020b), reducing stress (Huberty et al., 2019) and depression (Jones et al., 2022), improving patient safety competence levels (Liu et al., 2022a), and becoming a viable treatment option for anxiety disorders (Boettcher et al., 2014). Aside from these advantages, mindfulness began to play a greater role with high social value as scholars began to explore its application in other fields, and it has become a subject of interdisciplinary research (e.g., sociology, family studies, education, anthropology, philosophy, economics, and organizational science). However, the application of mindfulness in the field of communication is scarce. Social media is a popular topic in communication research, and it suggests new approaches to communicate across temporal and spatial boundaries (Whelan et al., 2020). Due to accessibility, recreation, and information approach, social media has prevailed all around the world (Chai and Kim, 2012). Nevertheless, social media is a double-edged sword, as studies have suggested that disproportionable and obsessive use of social media could result in users' psychological problems, such as fatigue (Ravindran et al., 2014), social anxiety (Yen et al., 2012), and depression (Twenge et al., 2019). As mindfulness may be a significant factor that could lead to diminishing of negative psychological emotions (Liu et al., 2020b), it is significant and necessary to explore its application in social media context.

### Effect of mindfulness on social media rumination

Rumination is the opposite of mindfulness (Heeren and Philippot, 2011). Rumination refers to the tendency to think repetitively about causes, situational factors, and consequences related to a stressful or distressing event (Nolen-Hoeksema and Morrow, 1993). Rumination has been recognized as a way of thinking negatively and is often related to low spirits, poor wellbeing, despair, and inefficiency (Broderick, 2005; Strizhakova et al., 2012; Javed et al., 2019). Despite the fact that researchers have connected rumination with depression, stress, and other psychological problems, researchers have rarely studied rumination in the context of social media, which is important due to the fact that use of social media is connected with mental distress as well as rumination about social media content in the same trend (Parris et al., 2020). Social media rumination refers to the situation under which one tends to think repetitively about his social media posts, other relevant situational factors, and consequences (Parris et al., 2020). Social media rumination has been linked to behavioral problems and negative psychological emotions in studies. As an example, a study found that social media rumination is strongly related to problematic smartphone use (Elhai et al., 2018), and that it may also aggravate symptoms of depression (Espelage et al., 2018). Therefore, it is important to explore factors that reduce social media rumination for social media users' mental health.

The drawback of rumination is clear, but research studies focusing on the leading ameliorating factor of social media rumination are still scant. As more and more people become engrossed in digital spaces, it is necessary to explore the factors affecting social media rumination to improve the situation. Social media users may feel social pressure when displaying themselves, leading to cautiously curating their posts; concerning about these, online self-presentation may draw out social media rumination (Feinstein et al., 2014; Yang et al., 2018). On the contrary, this study suggests that people who pay less attention to others and rarely rethink about their social media posts are less likely to elicit social media rumination. As a type of an individual's characteristic, mindfulness allows people to concentrate their cognitive attention to the present moment (Keng et al., 2011), thus making them less likely to rethink what they have posted online in the past and more likely to de-emphasize negative thoughts (Frewen et al., 2008). Mindfulness may help reduce rumination by balancing a wandering mind, where “wandering” can be repetitive thinking (Tingaz and Cakmak, 2021). The mitigating effect of mindfulness on rumination has been confirmed by numerous studies, and the current study suspects that a similar mechanism will also exist in the context of social media (Selby et al., 2016). Therefore, this article proposed the following hypothesis.

H1: mindfulness is negatively related to social media rumination.

### Upward social comparison as a mediator

Social comparison theory suggests that people have a tendency to compare their beliefs and talents to those of others. According to the tendency to choose what kind of people to compare, social comparisons are classified into three types: downward comparisons (comparing with people inferior to themselves), parallel comparisons (comparing with people similar to themselves), and upward comparisons (comparing with people superior to themselves) (Festinger, 1954). Considering that looking at others' profiles on social media platforms has already been a normal activity, social network sites seem to be a platform through which individuals could make social comparison (Pempek et al., 2009). A previous research study has found that users of social media platforms like Facebook, Twitter, and Instagram are more likely to believe that other users have higher social status than they do, leading to upward social comparisons (Latif et al., 2021). Upward social comparison on social media could also result in depression and social anxiety (Shaw et al., 2015), which are thought to be related to rumination (Strizhakova et al., 2012). Therefore, it is very important for social media users to make the negative effects brought about by upward social comparison diminish to keep their mental health in a good state.

The current study hypothesizes that upward social comparison is an important factor in causing ruminative thoughts on social media platforms, and that mindfulness mitigates rumination by reducing upward social comparison. In social media, upward social comparison has been shown to lead to some negative emotions, as users looking at others' photos and posts may enhance their self-perception of deficiencies that may lead to a negative feeling about themselves (Kim and Park, 2016). There is also evidence proving that upward social comparison is related to rumination. Considering the motivation for self-image management, users tend to present an idealized personal image on social media platforms in a positive way, which also means that they tend to spend a lot of time rethinking and decorating their social media posts (Jordan et al., 2011; Lee-Won et al., 2014). Rumination is a form of self-attention characterized by neurotic brooding and fixation on one's negative experiences (Nolen-Hoeksema et al., 2008). It is driven by perceived threats, losses, and injustices to the self, and social comparisons on social media can trigger these perceptions, which can easily lead to ruminative thoughts (Yang, 2022). Specifically, increased social media use could expose users to frequent upward social comparison, which leads to higher level of rumination (Vogel et al., 2014). Therefore, reducing upward social comparisons may be an effective path to reduce rumination. A previous study has shown that mindfulness is effective in reducing social comparison and its negative effects (Langer et al., 2010). Specifically, mindfulness suggests an approach to adopt a mindset of taking without judgments and to consider situations only with contemporary information. An experiment showed that mindfulness can help individuals better accept themselves without caring what others evaluate them to be, which leads to less upward comparison and the distress it brings (Wolsko, 2012). Previous research studies have discussed the relationship between upward social comparison and rumination, mindfulness and upward social comparison, and mindfulness and rumination; however, few studies have examined all three variables in one model (treating upward social comparison as a pathway for the influence of mindfulness on rumination). Based on the previous logic, mindfulness alleviates social media upward social comparison, and upward social comparison causes rumination, this article proposed the following hypothesis.

H2: upward social comparison mediates the relationship between mindfulness and social media rumination.

### Self-esteem as a moderator

Although mindfulness would negatively affect upward social comparison and rumination, the effects may be different for all individuals. A number of factors, particularly personal traits, may moderate the association between mindfulness and its results (Ford, 2017). Therefore, the effect of mindfulness on rumination may also be moderated by personal traits, and it is important to further identify these personal traits. Existing studies have explored the relationship between mindfulness and self-esteem. Many of them have confirmed a positive correlation between mindfulness and self-esteem (Gregoire et al., 2021; Rehman et al., 2021). However, few studies have considered self-esteem as a moderator between mindfulness and its influencing variables. Some researchers have noted this and have considered the moderating role of self-esteem while exploring the effects of mindfulness. A study on the impact of mindfulness on harmful responses to rejection noted that mindfulness only significantly helped individuals with low self-esteem and that those with high self-esteem did not benefit from mindfulness meditation (Ford, 2017). We therefore wondered whether self-esteem plays a similar role in the influence of mindfulness on upward social comparisons. Specifically, an individual's self-esteem level may moderate the effect of trait mindfulness on the individuals' negative psychology.

Self-esteem reflects a person's overall evaluation of her/his self-worth (Rosenberg et al., 1989); it is defined as the degree to which individuals evaluate themselves positively or negatively. Self-esteem is thought to be a fairly stable personality trait that differs between individuals (Waterman, 1992). It has been linked to a variety of behaviors in studies. Negative emotions and depression are less common in people who have a high self-esteem level than in people with a low level of self-esteem (Bandura, 1977). Self-esteem, as an important part of the self concept, is both an individual's temporary attitude and evaluation of self and a relatively stable personality trait that is gradually formed in the process of social interaction. It has an important impact on psychosocial adaptation for individuals (Leary, 2003). The sociometric theory of self-esteem suggests that self-esteem moderates individuals' perceptions and evaluation processes of others and external things (Leary, 2005). Social media is a convenient and efficient platform for social interaction and helps individuals with low self-esteem to cross interaction barriers, interact with others, and establish or maintain good interpersonal relationships (Lee et al., 2012). As a result, individuals with low self-esteem often treat social media as a substitute for offline social interaction, leading to more psychological problems and problematic online behaviors (Kuss and Griffiths, 2011). Nowadays, self-esteem has been catching the attention of social media researchers as it is deeply entangled with the social comparison process, playing the role as one of the key psychological phenomena related to social media use (Bayer et al., 2020).

Although the association between self-esteem and upward social comparison is well understood in social media contexts, we know little to determine factors that might be more effective in helping individuals with low self-esteem reduce upward social comparison. Preliminary evidence suggests that mindfulness can reduce an individual's upward social comparison (Langer et al., 2010). Trait mindfulness aims to focus attention and processing on the present moment in an accepting and nonjudgmental way. Individuals with a high level of mindfulness are better able to relate private events at the present moment in a positive light, manage complex emotions more effectively, and facilitate greater wellbeing in their daily lives (Apaolaza et al., 2019). As a result of not judging and avoiding criticism, mindfulness assists people in accepting themselves and improving their perceptions of who they are and their worth, which is very beneficial for individuals with low self-esteem (Pepping et al., 2013). With regard to individuals with low self-esteem, mindfulness may allow them to be less focused on social upward comparisons on social media. Conversely, individuals with high self-esteem are inherently less interested in upward social comparisons and therefore may benefit less from mindfulness (Ford, 2017). While much of the previous research has focused on direct effects of self-esteem, the current study aims to explore the moderating effects of self-esteem. The sociometer theory states that self-esteem is an indicator for individuals to evaluate their social relationship status, and that the higher the level of self-esteem, the better the individual's social relationship performance is likely to be. Social beliefs, social motivations, and social styles differ greatly among individuals with different levels of self-esteem (Anthony et al., 2007). It has been revealed that there are differences in attentional bias among individuals with different levels of self-esteem. Individuals with high self-esteem levels are more inclined to external attributions, and they tend to adopt positive defensive strategies to maintain high self-esteem; so when faced with upward social comparisons, they direct their attention to their own strengths and show positive coping styles, such as self-confidence and optimism. In contrast, individuals with low self-esteem prefer internal attributions, attribute their unfavorable social status to their own problems, and show negative emotions (Brown and Dutton, 1995). Many studies have confirmed the moderating effect of positive thinking on the outcome of other psychological trait influences (Wang et al., 2021). Based on this logic, we focus on differences in the performance of trait mindfulness across different self-esteem groups, although there may be a significant correlation between self-esteem and trait mindfulness. Since the effects of self-esteem and trait mindfulness are similar, we consider trait mindfulness to be more effective for individuals with low self-esteem. Specifically, the present study suggests that self-esteem plays a moderating role in the effect of trait mindfulness on upward social comparison. Despite previous studies on the relationship among mindfulness, self-esteem, and upward social comparison, few studies have attempted to test the interaction effect of self-esteem and mindfulness on upward social comparison. Self-esteem has been used as a moderator in several studies (Kraemer et al., 2001). Based on the above logic, we hypothesized that the level of self-esteem could moderate the effect of mindfulness on upward social comparison. Specifically, individuals who possess high self-esteem show less interest in upward social comparison. Therefore, these individuals may have less need for mindfulness to help reduce upward comparison on social media. Conversely, for social media users with low self-esteem, the mindfulness trait can effectively inhibit their willingness to make upward comparisons on social media. Based on the relationship among the three, this study proposed the following hypothesis:

H3: self-esteem moderates the relationship between mindfulness and upward social comparison.

The moderating effect of self-esteem may also appear simultaneously in the influence of upward social comparison on rumination. The behavioral plasticity theory suggests that individuals with low self-esteem are more susceptible to stress and therefore more reactive to stress because of upward social comparison sensitivity (Brockner, 1988). If upward social comparison is considered a stressor, high self-esteem is a protective factor that can help buffer the negative effects of stressor and thus reduce the risk of negative thinking (Hui et al., 2022). Moreover, self-esteem serves as a buffering factor that convinces individuals that differences in input standards are remediable (Wu and Pooler, 2014). Therefore, individuals with high-esteem are less likely to be threatened by upward social comparison and are less likely to suffer from rumination. Based on the above logic, this article proposes the following research hypothesis:

H4: self-esteem moderates the relationship between upward social comparison and social media rumination.

### Conceptual model

**Figure 1:**
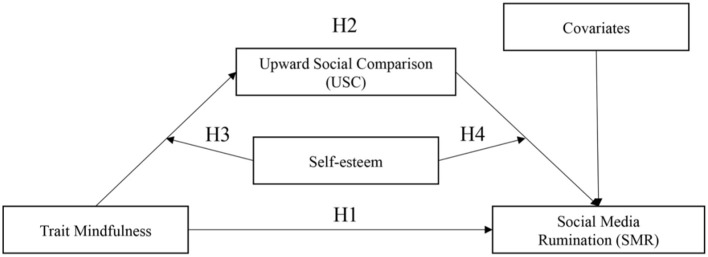
Conceptual model.

Based on the information above, our research developed a moderated mediation model (refer to Figure 1). The purpose of the model is to show how (the mediator role of upward social comparison) and when (the moderator role of self-esteem) a high level of mindfulness leads to a lower level of social media rumination.

## Materials and methods

### Study design

In order to ensure that our samples have an experience in using social media, we recruit participants through social media platforms. At the same time, we had to confirm that the participants are over 18 years old. Our study was a cross-sectional one to test the relationship between their mindfulness traits, self-esteem, upward social comparison, and social media rumination.

### Participants and procedures

“Questionnaire Star” (www.wjx.cn), a professional survey distribution platform, is chosen to be the approach for this study to collect questionnaires. We limited the IP address of the answering device so that every participant could not fill in the questionnaire twice. Meanwhile, our criteria for selecting the samples are participants over 18 years old who have an experience in using social media (if a respondent answers no in the two questions, the survey will be ended). In addition, we also set up identification questions, and questionnaires that failed to pass the identification questions were invalidated. With the aim of ensuring that the questions were clearly worded, the questionnaire was pretested in a small group of participants. The participants were informed of their right to withdraw, confidentiality, and anonymity prior to taking the survey. Computers, tablets, and mobile phones were all used to complete the cross-sectional survey. After consent was given, the participants began to answer our formal questionnaires. We collected a total of 380 questionnaires; after screening out invalid questionnaires (failed to answer the screening questions correctly and the answer time was <100 s), the final sample consisted of 317 current social media users.

A total of 380 questionnaires were collected. With an 83.4 % qualification rate, 63 questionnaires with unqualified answers, less time spent, and inconsistencies were eliminated. Men account for 48.6% of the sample, while women account for 51.4%. The sample's age ranged from 27 to 40 years old, with 65% falling into this category. In terms of education, the sample is concentrated on bachelor's degree holders, with a proportionate of 58.7%. In terms of daily social media usage, 96.2 % of the sample uses it for more than an hour. In Table 1, the basic demographic variables are tabulated.

**Table 1:** Statistical table of basic information on effective samples.

**Statistical items**	**Specific content**	**Statistical value**	**Percentage**
Gender	Male	154	48.6%
	Female	163	51.4%
Age	18–26	88	27.8%
	27–40	206	65.0%
	41–55	15	4.7%
	Over 55	8	2.5%
Educational	High School	14	4.4%
background	Undergraduate	186	58.7%
	Master and Doctor	117	36.9%
Social media usage	<1 h/ day	12	3.8%
duration	1–3 h/ day	116	36.6%
	3~5 h/ day	107	33.8%
	5 h/ day	82	25.9%

### Measurements

The instrument of this study included measures of mindfulness, upward social comparison, social media rumination, and self-esteem. The questionnaire of this study is developed following the pre-validated scales. Sub-items within each scale were averaged, resulting in composite scales. In consideration of the overall data analysis, the questionnaire adopts the Likert 7-point for all the scales. We also noticed that some of the original scales used even numbers; this may cause participants with neutral attitudes to choose options that are not appropriate for them, while odd numbered scales can solve this problem.

#### Trait mindfulness

The Trait Mindfulness Questionnaire is a 5-item self-reported scale, and items are adapted from the “act with awareness” dimension of the Five-Facet Mindfulness Questionnaire (Baer et al., 2006) (e.g., “I tend to be absent-minded and easily distracted when I do things”). It should be noted that, Turel adapted five items stemming from the act with awareness dimension of the Five-Facet Mindfulness Questionnaire to the context of social media use (Turel and Osatuyi, 2017). The validity of the five-item scale has also been verified in a previous study (Apaolaza et al., 2019). The participants were given a 7-point scale ranging from 1 (not at all true of me) to 7 (extremely true of me), with higher scores indicating higher levels of mindfulness. Cronbach's α was 0.838 in our study.

#### Upward social comparison

The Upward Social Media Comparison Questionnaire for this study is based on Gibbons et al.'s upward social comparison scale adapted in a social media context (Gibbons and Buunk, 1999). It is a 6-item questionnaire that assesses the level of upward social comparison among social media users (i.e., “I like to compare with those who live better than me on social media platforms”). A Likert scale of 1 (not at all true of me) to 7 (extremely true of me) was used to grade all the responses, with higher scores indicating more upward social comparisons. Cronbach 's α was 0.928 in this study.

#### Social media rumination

The Social Media Rumination Questionnaire was used to assess social media rumination, and the original scale included 12 items (Parris et al., 2020). We first excluded items for symptom rumination because of its high overlap with depressive symptoms (Treynor et al., 2003). In our pre-test, we merged the items that the participants perceived to be similar, because many of the participants commented that our overall questionnaire was too long, and that this made them feel fatigued. Therefore, we finally selected five items with a factor loading index higher than 0.6. It is a five-item self-reported survey (i.e., “do I worry about how people will react to my social media posts”). The responses were graded on a seven-point scale ranging from 1 (not at all true of me) to 7 (extremely true of me), with higher scores indicating more social media ruminations. Cronbach's α was 0.867 in our study.

#### Self-esteem

Our self-esteem scale is derived from the six-item version of the Rosenberg Self-Esteem Scale (Rosenberg et al., 1989; Apaolaza et al., 2019). After translating the scale into Chinese, we merged some similar expressions and eventually retained three of them (including three perspectives: state, cognition, and attitude), and all the factor loading indexes are higher than 0.6. In addition, it is a self-reported survey (e.g., “I have a positive attitude toward myself”). The responses were graded on a seven-point scale ranging from 1 (strongly disagree) to 7 (strongly agree), with higher scores indicating higher levels of self-esteem. Cronbach's α was 0.916 in this study.

### Data analysis

The validity and reliability of our questionnaire were tested using AMOS 26.0. The PROCESS macro of SPSS was used to evaluate the moderated mediation model with bootstrapping (95 % CI, 5,000 samples). Gender (0 = female, 1 = male), highest degree attained, age, and daily social media usage length are among the covariates examined in this model.

## Results

Before the data analysis, we checked for missing values and found none in the dataset.

### Measurement of the model

Table 2 shows that the Cronbach's α and composite reliability of the scales are higher than the acceptable value (>0.8). This means that the reliability is satisfactory. The CR values of all the variables ranged from 0.841 to 0.929 and were higher than the standard value (>0.7), indicating that the reliability of the combination of variables met the requirements (Hair et al., 2019). To assess for convergent validity, the AVE of the variables is calculated, with all the values above the allowed value (>0.5), showing positive convergent validity (Hair, 2010). Discriminant validity was tested by comparing the square root of AVE with the correlations of the researched variables. The square root of the AVE was greater than the correlations, indicating good discriminant validity (Fornell and Larcker, 1981).

**Table 2:** Results of validity and reliability.

	**1**	**2**	**3**	**4**	**AVE**	**CR**	**Cronbach's α**
1.USC	**0.851**				0.724	0.929	0.928
2.SMR	0.494	**0.771**			0.594	0.875	0.867
3.Self-esteem	−0.050	0.082	**0.885**		0.784	0.916	0.916
4.Mindfulness	−0.256	−0.153	0.062	**0.720**	0.518	0.841	0.838

The bold numbers on the diagonal represent the square root of AVE, AVE = Average Variance Extracted, CR = Construct Reliability.

The goodness of fit metrics was then evaluated. The confirmatory factor analysis (CFA) of our questionnaire produced satisfactory fit values for the one-dimensional factor structure after including the error covariances in the model (χ^2^/df = 2.528 < 0.3, RMSEA = 0.07 < 0.15, SRMR = 0.058 < 0.05, GFI = 0.915 > 0.9, CFI = 0.945 > 0.9, NFI = 0.912 > 0.9, and IFI = 0.945 > 0.9).

### Statistics

Table 3 shows the descriptive statistics and correlation analysis results. Mindfulness was negatively associated with upward social comparison (USC) and social media rumination (SMR). Upward social comparison (USC) was positively correlated with social media rumination (SMR).

**Table 3:** M, SD, and correlations among the variables.

**Research variables**	**M**	**SD**	**1**	**2**	**3**	**4**	**5**	**6**	**7**
1. Gender (male = 1 female = 2)									
2. Age			−0.096						
3. Highest Degree			0.091	−0.013					
4. Social Media Usage Duration			0.152^**^	−0.162^**^	0.112^*^				
5. Mindfulness	5.601	0.758	−0.086	0.230^**^	−0.126^*^	−0.034			
6. Self-esteem	4.849	1.361	0.113^*^	0.074	0.221^**^	0.019	0.062		
7. USC	3.121	1.542	0.067	−0.302^**^	0.041	0.181^**^	−0.256^**^	−0.050	
8. SMR	3.611	1.509	0.047	−0.286^**^	0.198^**^	0.282^**^	−0.153^**^	0.082	0.494^**^

* p <0.05;

** p <0.01.

### Relationship between mindfulness and social media rumination

We conducted a polynomial regression analysis using the PROCESS macro of SPSS, and the results are shown in Table 4. After controlling for highest degree obtained, gender, age, and daily social media usage duration, mindfulness significantly negatively affected the levels of upward social comparison (β = −1.287, SE = 0.375, t = −3.435, *p* = 0). Upward social comparison positively predicted social media rumination (β = 0.374, SE = 0.168, t = 2.23, *p* = 0.026) significantly. Although mindfulness and social media rumination were significantly negatively correlated in our survey (β = −1.292, *p* = 0.006), mindfulness had no significant direct effect on social media rumination (β = −0.011, SE = 0.1, t = −0.107, *p* = 0.915) in this model, H1 partly held. However, this will not affect the following analysis of moderated mediation, because whether mindfulness has an effect on social media rumination is not a prerequisite for moderated mediation. The SPSS PROCESS Model 4 Bootstrap test revealed that upward social comparison had a completely mediating effect of mindfulness on social media rumination [95% boot CI = (−0.139, −0.03)]. H2 held.

**Table 4:** Moderated mediation model's multiple regression results.

**Independent variable**	**β**	**SE**	**t**	**p**	**R^2^**	**F**
Dependent variable: Social media rumination (SMR)
Gender	−0.019	0.143	−0.131	0.896	0.576	19.152^***^
Age	−0.330	0.121	−2.723	0.007^**^		
Highest degree	0.409	0.129	3.178	0.002^**^		
Social media usage duration	0.308	0.084	3.691	0.003^**^		
Mindfulness	−0.011	0.100	0.107	0.915		
USC	0.374	0.168	2.230	0.026^*^		
USC × Self-esteem	0.008	0.033	0.233	0.816		
Dependent variable: Upward social comparison (USC)
Gender	−0.013	0.163	−0.079	0.937	0.406	8.693^***^
Age	−0.608	0.134	−4.526	0.000^***^		
Highest degree	0.046	0.147	0.313	0.754		
Social media usage duration	0.265	0.094	2.812	0.005^**^		
Mindfulness	−1.287	0.375	−3.435	0.000^***^		
Self-esteem	−1.155	0.457	−2.530	0.012^*^		
Mindfulness × Self-esteem	0.191	0.077	2.489	0.013^*^		

*** p <0.001,

** p <0.01, and

* p <0.05.

The Bootstrap test was performed using Model 58 in the SPSS PROCESS macro to assess the self-esteem moderation effect. Before creating the interaction term, mindfulness, upward social comparison, and self-esteem were concentrated. The analysis indicated that self-esteem significantly moderated the effect of mindfulness on upward social comparison (*p* = 0.013), implying that the moderated mediation model was established and that H3 was supported. Furthermore, the mediating impact was significant at low (−1 SD; 95 percent boot CI = (−0.415, −0.096)) and medium levels (95 percent boot CI = (−0.264, −0.049)) of self-esteem but not significant at high levels (+1 SD; 95 percent boot CI = (−0.209, 0.11)) of self-esteem. However, self-esteem cannot significantly moderate the effect of upward social comparison on social media rumination (*p* = 0.816). Therefore, H4 is not valid.

**Figure 2:**
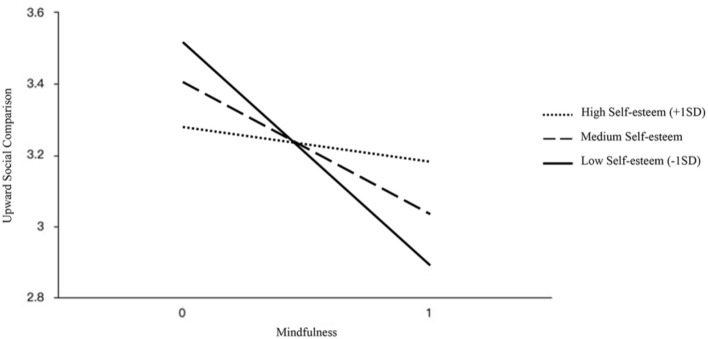
Interaction between mindfulness and self-esteem on the USC is tested by a simple slope test.

Meanwhile, the mindfulness × self-esteem interaction significantly predicted upward social comparison (β = 0.142, SE = 0.076, t = 2.503, *p* = 0.013 < 0.05; refer to Figure 2). Mindfulness had no significant effect on upward social comparison when the level of self-esteem was high (β = −0.104, SE = 0.161, t = −0.644, *p* = 0.52) and was significant when self-esteem is at medium (β = −0.364, SE = 0.111, t = −3.285, *p* = 0.001) and low (β = −0.624, SE = 0.143, t = −4.374, p = 0) levels.

## Discussion

**Figure 3:**
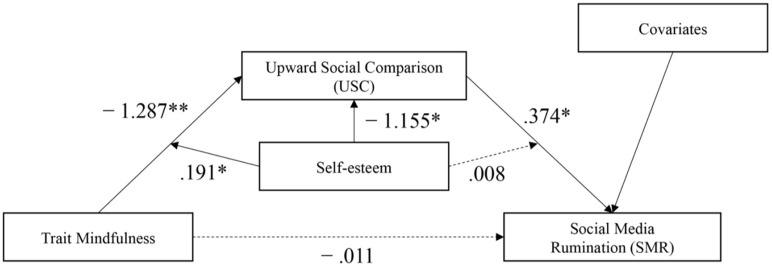
Moderated mediation model. Dashed lines represent nonsignificant relation. **p < 0.01 and *p < 0.05.

The aim of the present study was to explore the relationship among mindfulness, social media rumination, upward social comparison and self-esteem. It is exhibited that upward social comparison plays a mediating role, whereas self-esteem plays a moderating role (as shown in Figure 3). This help to clarify the process of how and under which situation the mindfulness of social media users affect their social media ruminations so as to provide a mechanism for dealing with individuals' psychological problems caused by social media.

### Mediation of upward social comparison

This study found that mindfulness has a significantly negative effect on upward social comparison, indicating that the higher level of mindfulness an individual possesses, the less likely he would perform upward comparison on social media, which is consistent with previous research results (Park et al., 2021). Meanwhile, upward social comparison can significantly predict social media rumination. The higher intensity an individual possesses toward upward social comparison, the higher risk of social media rumination he would possess, in line with previous research studies (Yang et al., 2018). In addition, the results of this study showed that upward social comparison completely mediated the effect of mindfulness on social media rumination. Specifically, in the correlation test, mindfulness was negatively correlated with social media rumination, but when we included upward social comparison as a mediating variable, the correlation between mindfulness and social media rumination was no longer significant. In social media context, the mitigating effect of mindfulness on social media rumination is mainly achieved by reducing upward social comparisons.

The use of social media enables users to learn about others' lives easily but also leads to upward social comparison (Wang et al., 2020). This research suggests that upward social comparison is strongly mediated by the relationship between mindfulness and social media rumination, and further explains the process under which mindfulness affects social media rumination; that is, mindfulness affects the social media rumination of social media users indirectly through upward social comparison, which is considered as a bridge between mindfulness and social media rumination.

### Moderation of self-esteem

Self-esteem moderates the relationship between mindfulness and upward social comparison but does not moderate the relationship between mindfulness and social media rumination, and that between upward social comparison and social media rumination. Enhancement of mindfulness does not make the upward social comparison of social media users with high self-esteem diminish significantly. It does, however, safeguard those with low and medium levels of self-esteem. That is to say, with enhancement of mindfulness, individuals with low and medium self-esteem make upward social comparison diminish significantly. This also clearly shows that self-esteem plays a buffering impact (which reduces low mindfulness individuals' upward comparing inclination), which is consistent with earlier findings (Pepping et al., 2013; Ford, 2017). The results of this study exhibit that individuals with low mindfulness is likely to tend to make an upward social comparison if their self-esteem level is not high. Therefore, for this group, raising the level of mindfulness could be effective in curbing upward social comparisons.

Meanwhile, the moderating role of self-esteem in the mediating process of “mindfulness–upward social comparison–social media rumination” has been demonstrated in this study. The results show that with decline in self-esteem, mindfulness influences the level of social media rumination through upward social comparison's mediating effect. Thus, social media users with low and medium self-esteem can avoid social media rumination by the similar approach as intervention of upward social media comparison (e.g., mindfulness training). However, this approach is not applied to individuals with high self-esteem, so other interventions should be sought.

### Effect of age and highest degree

As shown in Tables 3, 4, we found that age was correlated with mindfulness, USC, and SMR, and that highest degree was correlated with mindfulness, self-esteem, and SMR. First of all, in terms of age, although different scholars have different statements on the concept of generation, the consensus among them is that a generation will be influenced by the culture, society, and politics of the time period; thus, groups born in a similar time period will have similar perceptions and behaviors, while there are some stable differences among groups in different times. A number of studies have empirically demonstrated differences in the values and behaviors of different age groups in different research fields (Smola and Sutton, 2002). We therefore first tested the model using age as a moderator:

Mindfulness^*^Age → USC: β = −0.259, t = −1.578, *p* =0.116 [95% boot CI = (−0.582,0.64)]; USC^*^Age → SMR: β = 0.084, t = 1.072, *p* = 0.284 [95% boot CI = (−0.698,0.237)]. It was found that age did not play a moderating role in this model; however, age directly affected mindfulness, USC, and SMR. Specifically, age was positively associated with mindfulness, implying that older social media users had higher levels of trait mindfulness. In addition, age also negatively affected USC (β = −0.33, *p* = 0.007) and SMR (β = −0.608, *p* = 0), suggesting that older groups were less likely to prefer upward social comparison and have a lower level of rumination. This also means that the younger group may have more serious online mental health problems than the older group. Second, in terms of highest degree, we also conducted a test with highest degree as a moderator: Mindfulness^*^HD → USC: β = −0.11, t = −0.605, *p* = 0.545 [95% boot CI = (−0.468, 0.248)]; USC^*^HD → SMR: β = 0.019, t = 0.229, *p* = 0.545 [95% boot CI = (−0.149, 0.188)]. It was found that highest degree also did not play a moderating role in our model. In addition, we found that mindfulness was negatively correlated with highest degree, meaning that groups with higher education had lower levels of mindfulness instead. Moreover, as highest degree increased, individuals had more severe ruminations and higher self-esteem level. This finding is very revealing, as although some literature has confirmed the influence of academic qualifications on individuals' psychological traits (Karatas, 2015), few studies have explored the relationship between academic qualifications and rumination and upward social comparison, so future research could dig on this deeper.

### Implication

From a theoretical perspective, the present study suggests that people with low mindfulness and low self-esteem might have a risk of making upward social comparisons, which in turn may cause social media rumination, which may be harmful to their mental health. Moreover, self-esteem exhibits no increasing effect; but as a buffer, it makes social media users with low mindfulness less likely to make upward comparisons. Then, the degree of mediating effect of upward social comparison is different because of different levels of self-esteem. The mediating effect was not significant on social media users who have a high level self-esteem but significant in those with medium and low levels of self-esteem. These provide more comprehensive knowledge of understanding the social media rumination symptoms of social media users.

Considering the practical way, the current study reveals insights into under what conditions and how mindfulness can lead to lower social media rumination level, enabling feasible implications to reduce the risk of rumination of social media users with potential mental health problems: (a) upward social comparison is the risk factor of social media rumination for social media users. Therefore, mindfulness training is necessary for social media users to reduce upward social comparisons, especially for individuals with low and medium self-esteem. (b) More clarified suggestions can be provided considering the differences in personality characteristics and self-esteem of social media users. On one hand, improving their self-esteem according to the moderating effect can lower the possibility of making upward social media comparisons of individuals with low mindfulness. On the other hand, individuals with low self-esteem and mindfulness need to focus on contemporary to avoid judgments and being influenced by situational factors.

### Limitations and future directions

Although research on the topic of mindfulness and rumination is popular, especially in communication field, a large amount of research questions still remains unanswered. The present study, although it explores some valuable findings, has some deficiencies. To begin with, in our study, mindfulness refers to a wide term that is not divided into separate elements. It has been suggested that mindfulness is constructed by many facets or dimensions (Bishop et al., 2004; Coffey et al., 2010). The current study only focuses on the “act with awareness” dimension of mindfulness, so more dimensions of mindfulness can be taken into account in future studies. A two-component model has been proposed by Bishop including self-regulation of attention and orientation toward one's experience in the present. Future research could subdivide mindfulness to analyze whether different aspects would set a different impact on the relationship between social media rumination and upward social comparison. Second, in this study, mindfulness was measured *via* self-report; as a result, it recorded people's perceptions of their own trait mindfulness, which may have flaws, such as bias stemming from their acquaintance with mindfulness-related ideas (Grossman and Van Dam, 2011). However, when compared to other research using self-report measures, this study is not limited by them. Third, due to the limitation of translation, we modified some measurement items of their original scales, and although the modified scales have been tested for reliability and validity, this may challenge the external validity of our scale. Fourth, the data used in this study are cross-sectional and do not permit the testing of causal relationships. Fifth, this study only explored upward social comparison as a mediator; however, there are other influences on rumination [e.g., FOMO (Elhai et al., 2020), social media fatigue (Ye et al., 2020), and cyberbullying (Liu et al., 2020c)] that could be included in future studies. Sixth, the correlation analysis (Figure 3) shows that self-esteem is neither significantly correlated with trait mindfulness nor with upward social comparison in our study, which is inconsistent with previous research (Dion et al., 2021), probably because we measured only trait mindfulness. Future research could explore more the correlation between other dimensions of mindfulness and self-esteem. Finally, although the current research study included a diverse sample, we did not examine the influence of gender, generation, and social media usage as key study variables. Investigating gender and generational disparities could help develop more effective therapies for reducing social media rumination. They should be considered in future investigations.

## Conclusion

This research focuses on the rumination of social media users and aims to provide insights into how (the role of upward social comparison as a mediator) and under what situations (the role of self-esteem as a moderator) mindfulness can reduce the level of social media rumination. Study finds that those social media users with low mindfulness and low or medium self-esteem may have an upward social comparison tendency. However, self-esteem reduces the danger of upward social comparison in individuals with low mindfulness, and upward social comparison only has a mediating influence on people with medium or low self-esteem. The insights from this study may help to have a better understanding of the rumination of social media users and provide more feasible intervention suggestions to improve the situation.

## Data availability statement

The original contributions presented in the study are included in the article/Supplementary material, further inquiries can be directed to the corresponding author.

## Ethics statement

This study was reviewed and approved by the academic committee of the school of Journalism and communication of Xiamen University. Written informed consent from the patients/participants was not required to participate in this study in accordance with the national legislation and the institutional requirements.

## Author contributions

CG is responsible for the overall research design, thesis writing, collation of the questionnaires, and data analysis. SC is responsible for the guidance. SL is responsible for the proofreading and article touch up. All the authors in the research team have contributed to the thesis. All authors contributed to the article and approved the submitted version.

## Conflict of interest

The authors declare that the research was conducted in the absence of any commercial or financial relationships that could be construed as a potential conflict of interest.

## Publisher's note

All claims expressed in this article are solely those of the authors and do not necessarily represent those of their affiliated organizations, or those of the publisher, the editors and the reviewers. Any product that may be evaluated in this article, or claim that may be made by its manufacturer, is not guaranteed or endorsed by the publisher.
